# Some complementary inequalities to Jensen’s operator inequality

**DOI:** 10.1186/s13660-018-1616-z

**Published:** 2018-01-25

**Authors:** Jadranka Mićić, Hamid Reza Moradi, Shigeru Furuichi

**Affiliations:** 1 Faculty of Mechanical Engineering and Naval Architecture, University of Zagreb, Zagreb, Croatia; 2 Young Researchers and Elite Club, Mashhad Branch, Islamic Azad University, Mashhad, Iran; 3 Department of Information Science, College of Humanities and Sciences, Nihon University, Tokyo, Japan

**Keywords:** 47A63, 47A64, 47B15, 46L05, self-adjoint operator, positive linear mapping, convex function, converse of Jensen’s operator inequality, Mond-Pečarić method

## Abstract

In this paper, we study some complementary inequalities to Jensen’s inequality for self-adjoint operators, unital positive linear mappings, and real valued twice differentiable functions. New improved complementary inequalities are presented by using an improvement of the Mond-Pečarić method. These results are applied to obtain some inequalities with quasi-arithmetic means.

## Introduction

Let $\mathcal{B}(\mathcal{H})$ (resp. $\mathcal{B}_{h}(\mathcal{H})$) be the set of all bounded linear operators (resp. all self-adjoint operators) on a Hilbert space $\mathcal{H}$. A real-valued continuous function *f* defined on an interval *I* is said to be operator convex if $f(tA+(1-t)B) \leq tf(A)+(1-t)f(B)$ for all $t \in[0,1]$ and all self-adjoint operators $A,B$ in $\mathcal{B}_{h}(\mathcal{H})$ with spectra contained in *I*. We recall the Davis-Choi-Jensen inequality (so-called Jensen’s operator inequality): $f(\Phi(A)) \leq\Phi (f(A))$ for every self-adjoint operator *A* with $m \leq A\leq M$, where *f* is an operator convex function on an interval $[m,M]$, and $\Phi: \mathcal{B}(\mathcal{H}) \mapsto\mathcal{B}(\mathcal{K})$ is a unital positive linear mapping (see [1–3]).

Jensen’s inequality is one of the most important inequalities. It has many applications in mathematics and statistics, and some other well-known inequalities are its particular cases. There is an extensive literature devoted to Jensen’s operator inequality regarding its variuous generalizations, refinements, and extensions; see, for example, [3–9].

On the other hand, Mond and Pečarić [10, 11] (also see [12, 13]) investigated converses of Jensen’s inequality. To present these results, we introduce some abbreviations. Let $f:[m,M]\rightarrow\mathbb{R}$, $m< M$. Then a linear function through $(m,f(m))$ and $(M,f(M))$ has the form $h(z)=k_{f} z + l_{f}$, where

1 $$ k_{f}:=\frac{f(M)-f(m)}{M-m}\quad \text{and}\quad l_{f}:=\frac{Mf(m)-mf(M)}{M-m}. $$

Using the Mond-Pečarić method, a generalized complementary inequality of Jensen’s operator inequality is presented in [14]. A continuous version of this inequality is presented in [15]. Also, Mićić, Pavić, and Pečarić [16] obtained a better bound than that in [15] under the assumptions that $(x_{t})_{t\in T}$ is a bounded continuous field of self-adjoint elements in a unital $C^{*}$-algebra ${\mathcal {A}}$ with spectra in $[m,M]$, $m< M$, defined on a locally compact Hausdorff space *T* equipped with a bounded Radon measure *μ*, and $(\phi_{t})_{t\in T}$ is a unital field of positive linear mappings $\phi_{t} :{\mathcal {A}}\to {\mathcal {B}}$ from ${\mathcal {A}}$ to another unital $C^{*}$-algebra ${\mathcal {B}}$. *If* $m_{x}$ *and* $M_{x}$*,* $m_{x}\leq M_{x}$*, are the bounds of the self-adjoint operator* $x=\int_{T}\phi_{t}(x_{t}) \,d\mu(t)$*,* $f:[m,M]\to \mathbb{R}$ *is convex,* $g:[m_{x},M_{x}]\to \mathbb{R}$*,* $F:U\times V\to \mathbb{R}$ *is bounded and operator monotone in the first variable, with* $f([m,M])\subseteq U$*, and* $g([m_{x},M_{x}])\subseteq V$*, then*

2 $$ F \biggl[ \int_{T}\phi_{t}\bigl(f(x_{t})\bigr) \,d \mu(t) , g \biggl( \int_{T}\phi_{t}(x_{t}) \,d\mu(t) \biggr) \biggr] \leq C_{1} 1_{\mathcal{K}} \leq C 1_{\mathcal{K}}, $$

*where*

$$\begin{aligned}& C_{1} \equiv C_{1} (F,f,g, m, M, m_{x}, M_{x}) = \sup_{m_{x} \leq z \leq M_{x}} F \bigl[k_{f} z + l_{f},g(z) \bigr], \\& C \equiv C (F,f,g, m, M) = \sup_{m \leq z \leq M} F \bigl[k_{f} z + l_{f},g(z) \bigr]. \end{aligned}$$

Moreover, Mićić, Pečarić, and Perić [7] obtained a refinement of (2). For convenience, we introduce abbreviations *x̃* and $\delta_{f}$ as follows:

3 $$ \tilde{x} \equiv\tilde{x}_{x_{t},\phi_{t}}(m,M):= \frac{1}{2} 1_{\mathcal{K}} - \frac{1}{M-m} \int_{T}\phi_{t} \biggl( \biggl\vert x_{t}- \frac {m+M}{2} 1_{\mathcal{H}} \biggr\vert \biggr) \,d \mu(t), $$

where *m*, *M*, $m < M$, are scalars such that the spectra of $x_{t}$ are in $[m,M]$, $t \in T$;

4 $$ \delta_{f} \equiv\delta_{f}(m,M) := f(m)+f(M) - 2 f \biggl( \frac {m+M}{2} \biggr), $$

where $f:[m,M]\rightarrow\mathbb{R}$ is a continuous function. Obviously, $\tilde{x}\geq0$, and $\delta_{f} \geq0$ for *convex* *f* or $\delta_{f} \leq0$ for *concave* *f*.

Under the above assumptions, they proved in [7] that

5 $$\begin{aligned}& F \biggl[ \int_{T}\phi_{t}\bigl(f(x_{t})\bigr) \,d \mu(t) , g \biggl( \int_{T}\phi_{t}(x_{t}) \,d\mu(t) \biggr) \biggr] \\& \quad \leq F \biggl[k_{f} \int_{T}\phi_{t}(x_{t}) \,d\mu(t) + l_{f} 1_{\mathcal{K}} - \delta_{f} \tilde{x} , g \biggl( \int_{T}\phi_{t}(x_{t}) \,d\mu(t) \biggr) \biggr] \\& \quad \leq \sup_{m_{x} \leq z \leq M_{x}} F \bigl[k_{f} z + l_{f} - \delta_{f} m_{\tilde{x}},g(z) \bigr] 1_{\mathcal{K}} \leq C_{1} 1_{\mathcal {K}} \leq C 1_{\mathcal{K}}, \end{aligned}$$

where $m_{\tilde{x}}$ is the lower bound of the operator *x̃*. More precisely, they obtained the following improved difference- and radio-type inequalities (for a convex function *f*):

6 $$\begin{aligned}& \int_{T}\phi_{t}\bigl(f(x_{t})\bigr) \,d \mu(t) \leq g \biggl( \int_{T}\phi_{t}(x_{t}) \,d\mu(t) \biggr) \\& \hphantom{\int_{T}\phi_{t}\bigl(f(x_{t})\bigr) \,d \mu(t)\leq{}}{}+ \max_{m_{x}\leq z\leq M_{x}} \bigl\{ k_{f} z + l_{f} - g(z) \bigr\} 1_{\mathcal{K}} - \delta_{f} \tilde{x}, \end{aligned}$$

7 $$\begin{aligned}& \int_{T}\phi_{t}\bigl(f(x_{t})\bigr) \,d \mu(t) \leq \max_{m_{x}\leq z\leq M_{x}} \biggl\{ \frac{k_{f} z + l_{f}}{g(z)} \biggr\} g \biggl( \int_{T}\phi_{t}(x_{t}) \,d\mu(t) \biggr) - \delta_{f} \tilde{x}, \end{aligned}$$

and

8 $$ \int_{T}\phi_{t}\bigl(f(x_{t})\bigr) \,d \mu(t) \leq \max_{m_{x}\leq z\leq M_{x}} \biggl\{ \frac{ k_{f} z + l_{f} - \delta_{f} m_{\tilde{x}} }{g(z)} \biggr\} g \biggl( \int_{T}\phi_{t}(x_{t}) \,d\mu(t) \biggr), $$

where $g>0$ on $[m_{x},M_{x}]$ in (7) and (8).

In this paper, we obtain some complementary inequalities to Jensen’s operator inequality for twice differentiable functions. We obtain new inequalities improving the same type inequalities given in [17]. In particular, we improve inequalities (5), (7), and (8). Applying the obtained results, we give some inequalities for quasi-arithmetic means.

## Some auxiliary results without convexity

In this section, we give some results, which we will use in the next sections.

To prove our next result, we need the following lemma.

### Lemma A

([7])

*Let* *f* *be a convex function on an interval* *I*, $m, M \in I$, *and* $p_{1}, p_{2} \in[0, 1]$ *such that* $p_{1} + p_{2} = 1$. *Then*

9 $$ \min\{p_{1}, p_{2} \} \delta_{f} \leq p_{1} f (m) + p_{2} f (M) - f (p_{1} m + p_{2} M) \leq\max\{p_{1}, p_{2} \} \delta_{f} , $$

*where* $\delta_{f} := f(m)+f(M) - 2 f ( \frac{m+M}{2} )$.

### Proof

These results follow from [18, Theorem 1, p. 717] for $n=2$. For the reader’s convenience, we give an elementary proof.

Since *f* is convex, we have

10 $$ f \biggl(\frac{\alpha x +\beta y}{\alpha+\beta} \biggr) \leq\frac {\alpha f(x) +\beta f(y)}{\alpha+\beta} $$

for all $x,y \in I$ and positive weights *α*, *β*.

Suppose that $0< p_{1}< p_{2}<1$, $p_{1} + p_{2} = 1$. Then, applying (10), we obtain

$$\begin{aligned}& 2 p_{2} f \biggl(\frac{p_{2}m + p_{2}M}{2 p_{2}} \biggr) -f(p_{1} m+p_{2} M) \\& \quad \leq(2 p_{2}-1) f \biggl(\frac{(p_{2}-p_{1})m+(p_{1}-p_{1})M}{2 p_{2}-1} \biggr) \\& \quad \leq (p_{2}-p_{1})f(m)+(p_{1}-p_{1})f(M) \\& \quad = p_{2} \bigl(f(m)+f(M)\bigr) - \bigl(p_{1} f(m)+p_{2}f(M)\bigr). \end{aligned}$$

It follows that

$$\begin{aligned}& p_{1} f(m)+p_{2}f(M)-f(p_{1} m+p_{2} M) \\& \quad \leq p_{2} \biggl( f(m)+f(M) - 2 f \biggl(\frac{m+M}{2 } \biggr) \biggr) = \max\{p_{1}, p_{2} \} \delta_{f}, \end{aligned}$$

which gives the second inequality in (9).

Also, applying (10), we obtain

$$\begin{aligned}& f(p_{1}m+p_{2}M) - 2 p_{1} f \biggl( \frac{p_{1}m + p_{1}M}{2 p_{1}} \biggr) \\& \quad \leq(1-2p_{1}) f \biggl(\frac{(p_{1}-p_{1})m+(p_{2}-p_{1})M}{1-2 p_{1}} \biggr) \\& \quad \leq (p_{1}-p_{1})f(m)+(p_{2}-p_{1})f(M) \\& \quad = p_{1} f(m)+p_{2}f(M) - p_{1} \bigl(f(m)+f(M) \bigr). \end{aligned}$$

It follows that

$$\begin{aligned}& p_{1} f(m)+p_{2}f(M)-f(p_{1} m+p_{2} M) \\& \quad \geq p_{1} \biggl( f(m)+f(M) - 2 f \biggl(\frac{m+M}{2 } \biggr) \biggr) = \min\{p_{1}, p_{2} \} \delta_{f}, \end{aligned}$$

which gives the first inequality in (9).

If $p_{1}=0$, $p_{2}=1$, or $p_{1}=1$, $p_{2}=0$, then inequality (9) holds, since *f* is convex. If $p_{1}=p_{2}=1/2$, then we have an equality in (9). □

Applying Lemma A, we obtain the following inequalities for twice differentiable functions.

### Lemma 1

*Let* *A* *be a self*-*adjoint operator with* $\sigma ( A )\subseteq [ m,M ]$ *for some* $m< M$, *let* $\Phi:\mathcal {B} ( \mathcal{H} )\to\mathcal{B} ( \mathcal{K} )$ *be a unital positive linear mapping*, *and let* $f:[m,M]\to \mathbb{R}$ *be a twice differentiable function*.

*If* $\alpha\le f''$ *on* $[m,M]$ *for some* $\alpha\in\mathbb{R}$, *then*

11 $$\begin{aligned} \Phi\bigl(f (A)\bigr) \leq& k_{f} \Phi(A) + l_{f} 1_{\mathcal{K}} -\frac{\alpha}{2} \bigl((M+m)\Phi(A) -mM 1_{\mathcal{K}} -\Phi \bigl(A^{2}\bigr) \bigr) \\ &{}- \biggl( \delta_{f} - \frac{\alpha}{4}(M-m)^{2} \biggr) \widetilde{A} \end{aligned}$$

*and*

12 $$\begin{aligned} \Phi\bigl(f (A)\bigr) \geq& k_{f} \Phi(A) + l_{f} 1_{\mathcal{K}}-\frac{\alpha}{2} \bigl( (M+m)\Phi(A) -mM 1_{\mathcal{K}} -\Phi \bigl(A^{2}\bigr) \bigr) \\ &{} - \biggl( \delta_{f} - \frac{\alpha}{4}(M-m)^{2} \biggr) (1_{\mathcal{K}}- \widetilde{A}), \end{aligned}$$

*where* $k_{f}$, $l_{f}$ *are defined by* (1), $\delta_{f}$ *is defined by* (4), *and*

13 $$ \widetilde{A} \equiv\widetilde{A}_{A,\Phi}(m,M):= \frac{1}{2} 1_{\mathcal{K}} - \frac{1}{M-m} \Phi \biggl( \biggl\vert A- \frac {m+M}{2} 1_{\mathcal{H}} \biggr\vert \biggr). $$

*If* $f'' \leq\beta$ *on* $[m,M]$ *for some* $\beta\in\mathbb{R}$, *then the reverse inequalities are valid in* (11) *and* (12) *with* *β* *instead of* *α*.

### Proof

We prove only the case $\alpha\le f''$. Using (9), we obtain

14 $$\begin{aligned}& p_{1} g (m) + p_{2} g (M) -\max \{p_{1}, p_{2} \} \delta_{g} \\& \quad \leq g (p_{1} m + p_{2} M) \leq p_{1} g (m) + p_{2} g (M)- \min\{ p_{1}, p_{2} \} \delta_{g} \end{aligned}$$

for every convex function *g* on $[m, M]$ and $p_{1}, p_{2} \in[0, 1]$, $p_{1} + p_{2} = 1$. For any $z \in[m,M]$, we can write

$$ z =\frac{M-z}{M-m}m+\frac{z-m}{M-m}M= p_{1}(z)m+p_{2}(z)M. $$

By (14) we get

15 $$ g (z) \leq \frac{M-z}{M-m} g (m) + \frac{z-m}{M-m} g (M) - \tilde{z} \delta_{g} = k_{g} z + l_{g} - \tilde{z} \delta_{g} $$

and

16 $$ g (z) \geq\frac{M-z}{M-m} g (m) + \frac{z-m}{M-m} g (M) - (1-\tilde{z}) \delta_{g} = k_{g} z + l_{g} - (1- \tilde{z}) \delta_{g} , $$

where we denote $\tilde{z} := \frac{1}{2} - \frac{1}{M-m} \vert z- \frac{m+M}{2} \vert $ and use the equalities

$$\begin{aligned}& \min \biggl\{ \frac{M-z}{M-m} , \frac{z-m}{M-m} \biggr\} = \frac {1}{2} - \frac{1}{M-m} \biggl\vert z- \frac{m+M}{2} \biggr\vert = \tilde{z}, \\& \max \biggl\{ \frac{M-z}{M-m} , \frac{z-m}{M-m} \biggr\} = \frac {1}{2} + \frac{1}{M-m} \biggl\vert z- \frac{m+M}{2} \biggr\vert = 1 - \tilde{z}. \end{aligned}$$

Next, since ${\sigma}(A)\subseteq[m,M]$, applying the functional calculus to (15) and (16), we obtain

17 $$ \Phi\bigl(g (A)\bigr) \leq k_{g} \Phi(A) + l_{g} 1_{\mathcal{K}} - \delta_{g} \widetilde{A} $$

and

18 $$ \Phi\bigl(g(A)\bigr) \geq k_{g} \Phi(A) + l_{g} 1_{\mathcal{K}} - \delta_{g} (1-\widetilde{A}) , $$

respectively. Since $g_{\alpha}(z)=f(z)-\frac{\alpha}{2}z^{2}$ is convex on $[m,M]$, (17) and (18) give

$$\begin{aligned} \Phi\bigl(f (A)\bigr) \leq&\frac{\alpha}{2} \Phi\bigl(A^{2}\bigr) + k_{f} \Phi(A) + l_{f} 1_{\mathcal{K}} -\frac{\alpha}{2} \bigl( (M+m)\Phi(A) - mM 1_{\mathcal{K}} \bigr) \\ &{}- \biggl( \delta_{f} - \frac{\alpha}{4} (M-m)^{2} \biggr) \widetilde{A} \end{aligned}$$

and

$$\begin{aligned} \Phi\bigl(f (A)\bigr) \geq&\frac{\alpha}{2} \Phi\bigl(A^{2}\bigr) + k_{f} \Phi(A) + l_{f} 1_{\mathcal{K}} -\frac{\alpha}{2} \bigl( (M+m)\Phi(A) - mM 1_{\mathcal{K}} \bigr) \\ &{}- \biggl( \delta_{f} - \frac{\alpha}{4} (M-m)^{2} \biggr) (1-\widetilde{A}), \end{aligned}$$

respectively, which give the desired inequalities (11) and (12). □

### Remark 1

(1) Observe that, in (11) and (12), $(M+m)\Phi(A) -mM 1_{\mathcal{K}} -\Phi(A^{2})\geq0$, $0\leq \widetilde{A}\leq\frac{1}{2}1_{\mathcal{K}}$, $\frac{1}{2}1_{\mathcal{K}} \leq1_{\mathcal{K}}-\widetilde{A} \leq 1_{\mathcal{K}}$, and $\delta_{f} - \frac{\alpha}{4}(M-m)^{2} \geq0 \geq\delta_{f} - \frac{\beta}{4}(M-m)^{2} $, since the functions $z \mapsto f(z)-\frac{\alpha}{2}z^{2}$ and $z \mapsto\frac{\beta}{2}z^{2} -f(z)$ are convex on $[m,M]$.

Then, (11) improves the first inequality in [17, Lemma 2.1], that is,

$$\begin{aligned} f \bigl(\Phi(A)\bigr) \leq& k_{f} \Phi(A) + l_{f} 1_{\mathcal{K}} -\frac{\alpha}{2} \bigl((M+m)\Phi(A) -mM 1_{\mathcal{K}} - \Phi \bigl(A^{2}\bigr) \bigr) \\ &{}- \biggl( \delta_{f} - \frac{\alpha}{4}(M-m)^{2} \biggr) \widetilde{A} \\ \leq& k_{f} \Phi(A) + l_{f} 1_{\mathcal{K}} - \frac{\alpha}{2} \bigl((M+m)\Phi(A) -mM 1_{\mathcal{K}} -\Phi \bigl(A^{2}\bigr) \bigr) . \end{aligned}$$

Also, the inequality with *β* improves the second inequality in [17, Lemma 2.1]:

$$\begin{aligned} f \bigl(\Phi(A)\bigr) \geq& k_{f} \Phi(A) + l_{f} 1_{\mathcal{K}} -\frac{\beta}{2} \bigl((M+m)\Phi(A) -mM 1_{\mathcal{K}} - \Phi \bigl(A^{2}\bigr) \bigr) \\ &{}- \biggl( \delta_{f} - \frac{\beta}{4}(M-m)^{2} \biggr) \widetilde{A} \\ \geq &k_{f} \Phi(A) + l_{f} 1_{\mathcal{K}} - \frac{\beta}{2} \bigl((M+m)\Phi(A) -mM 1_{\mathcal{K}} -\Phi \bigl(A^{2}\bigr) \bigr) . \end{aligned}$$

(2) Using (15), we get

19 $$\begin{aligned} f \bigl(\Phi(A)\bigr) \leq& k_{f} \Phi(A) + l_{f} 1_{\mathcal{K}} -\frac{\alpha}{2} \bigl((M+m)\Phi(A) -mM 1_{\mathcal{K}} -\Phi (A)^{2} \bigr) \\ &{}- \biggl( \delta_{f} - \frac{\alpha}{4}(M-m)^{2} \biggr) \hat{A}, \end{aligned}$$

where $\hat{A} := \frac{1}{2} 1_{\mathcal{K}} - \frac{1}{M-m} | \Phi(A)- \frac{m+M}{2} 1_{\mathcal{H}} | $. This inequality improves the third inequality in [17, Lemma 2.1]. Also, using (16), we get its complementary inequality

$$\begin{aligned} f \bigl(\Phi(A)\bigr) \geq& k_{f} \Phi(A) + l_{f} 1_{\mathcal{K}}-\frac{\alpha}{2} \bigl( (M+m)\Phi(A) -mM 1_{\mathcal{K}} -\Phi (A)^{2} \bigr) \\ &{} - \biggl( \delta_{f} -\frac{\alpha}{4}(M-m)^{2} \biggr) (1_{\mathcal{K}}-\hat{A}). \end{aligned}$$

(3) Combining (19) with the corresponding inequalities with *β* in Lemma 1, we can get improved inequalities [17, Theorem 2.1]. We omit the details.

## Main results

In this section, we generalize or improve some inequalities in Section 1 and [17].

Applying Lemma (1) and using the Mond-Pečarić method, we present versions of inequalities (2) and (5) without convexity and for one operator. We omit the proof.

### Lemma 2

*Let* *A*, Φ, *m*, *M*, $k_{f}$, $l_{f}$, $\delta_{f}$, *and* *Ã* *be as in Lemma *1, *and let* $m_{\Phi(A)}$ *and* $M_{\Phi (A)}$, $m_{\Phi(A)} \leq M_{\Phi(A)}$, *be the bounds of the self*-*adjoint operator* $\Phi(A)$. *Let* $f:[m,M]\to\mathbb{R}$ *be a twice differentiable function with* $f([m,M])\subseteq U$, $g:[m_{\Phi (A)},M_{\Phi(A)}]\to\mathbb{R}$ *be continuous with* $g([m_{\Phi (A)},M_{\Phi(A)}])\subseteq V$, *and* $F:U\times V\to \mathbb{R}$ *be bounded and operator monotone in the first variable*.

*If* $\alpha\le f''$ *on* $[m,M]$ *for some* $\alpha\in\mathbb{R}$, *then*

20 $$\begin{aligned}& F \bigl[\Phi\bigl(f(A)\bigr) , g \bigl(\Phi(A) \bigr) \bigr] \\& \quad \leq F \biggl[ k_{f} \Phi(A) + l_{f} 1_{\mathcal{K}} -\frac{\alpha}{2} \bigl((M+m)\Phi(A) -mM 1_{\mathcal{K}} - \Phi\bigl(A^{2}\bigr) \bigr) \\& \qquad {}- \biggl( \delta_{f} - \frac{\alpha}{4}(M-m)^{2} \biggr) \widetilde{A} , g \bigl(\Phi(A) \bigr) \biggr] \\& \quad \leq \sup_{m_{\Phi(A)} \leq z \leq M_{\Phi(A)}} F \biggl[ k_{f} z +l_{f}-\frac{\alpha(M+m)z -\alpha mM -M_{1} }{2} \\& \qquad {} - \biggl( \delta_{f} - \frac{\alpha}{4}(M-m)^{2} \biggr) m_{\widetilde{A}} ,g(z) \biggr] 1_{\mathcal{K}} \\& \quad \leq \sup_{m_{\Phi(A)} \leq z \leq M_{\Phi(A)}} F \biggl[ k_{f} z +l_{f}-\frac{\alpha(M+m)z -\alpha mM -M_{1} }{2} ,g(z) \biggr] 1_{\mathcal{K}}, \end{aligned}$$

*where* $M_{1}$ *is the upper bound of the operator* $\alpha\Phi(A^{2})$, $M_{1} \leq\max\{ \alpha m^{2}, \alpha M^{2} \} $, *and* $m_{\widetilde{A}}$ *is the lower bound of the operator* *Ã*.

*Further*,

21 $$\begin{aligned}& F \bigl[\Phi\bigl(f(A)\bigr) , g \bigl(\Phi(A) \bigr) \bigr] \\& \quad \geq F\biggl[k_{f} \Phi(A) + l_{f} 1_{\mathcal{K}} -\frac{\alpha}{2} \bigl((M+m)\Phi(A) -mM 1_{\mathcal{K}} -\Phi \bigl(A^{2}\bigr) \bigr) \\& \qquad {}- \biggl( \delta_{f} - \frac{\alpha}{4}(M-m)^{2} \biggr) (1_{\mathcal{K}}-\widetilde{A}) , g \bigl(\Phi(A) \bigr)\biggr] \\& \quad \geq \inf_{m_{\Phi(A)} \leq z \leq M_{\Phi(A)}} F \biggl[ k_{f} z +l_{f}-\frac{\alpha(M+m)z -\alpha mM -m_{1} }{2} \\& \qquad {}- \biggl( \delta_{f} - \frac{\alpha(M-m)^{2} }{4} \biggr) ( 1-m_{\widetilde{A}} ),g(z) \biggr] 1_{\mathcal{K}} \\& \quad \geq \inf_{m_{\Phi(A)} \leq z \leq M_{\Phi(A)}} F \biggl[ k_{f} z +l_{f}-\frac{\alpha(M+m)z -\alpha mM -m_{1} }{2} - \delta_{f} ( 1-m_{\widetilde{A}} ),g(z) \biggr] 1_{\mathcal{K}}, \end{aligned}$$

*where* $m_{1}$ *is the lower bound of the operator* $\alpha\Phi(A^{2})$, $m_{1} \leq\min\{ \alpha m^{2}, \alpha M^{2} \} $.

*However*, *if* $f'' \leq\beta$ *on* $[m,M]$ *for some* $\beta\in\mathbb {R}$, *then the reverse inequalities are valid in* (20) *and* (21) *with* inf *instead of* sup, sup *instead of* inf, *β* *instead of* *α*, $M_{1}$ *instead of* $m_{1}$, $m_{1}$ *instead of* $M_{1}$, *and* $M_{\widetilde{A}}$ *instead of* $m_{\widetilde {A}}$, *where* $M_{\widetilde{A}}$ *is the upper bound of the operator* *Ã*.

### Example 1

To illustrate Lemma 2, let $F(u,v)=u-v$ and $f(z)\equiv g(z)=z^{3}$ on $[m,M]$. Since $f''(z)=6z$, we can put $\alpha=6m$ and $\beta=6M$. Then $\delta_{f}=\frac{3}{4}(M-m)^{2}(m+M)$ (he sign which is not determined) and $\delta_{f} -\frac{\alpha}{4}(M-m)^{2} =\frac {3}{4}(M-m)^{3} \geq0$.

If $m<0< M$, then *f* is neither convex nor concave on $[m,M]$, and (6) is not in general valid:

22 $$\begin{aligned} \Phi\bigl(A^{3}\bigr) \nleq&\Phi(A)^{3} + \max _{m_{\Phi(A)} \leq z \leq M_{\Phi(A)}} \biggl\{ \frac{M-z}{M-m}m^{3}+ \frac{z-m}{M-m} M^{3} -z^{3} \biggr\} 1_{\mathcal{K}} \\ &{}- \frac{3}{4}(M-m)^{2}(m+M) {\widetilde{A}}, \end{aligned}$$

but applying the first inequality in (20), we have:

23 $$\begin{aligned} \Phi\bigl(A^{3}\bigr) \leq& \Phi(A)^{3} +\max _{m_{\Phi(A)} \leq z \leq M_{\Phi(A)}} \biggl\{ \frac{M-z}{M-m}m^{3}+ \frac{z-m}{M-m} M^{3} -z^{3} \biggr\} 1_{\mathcal {K}} \\ &{} -3m \bigl((M+m)\Phi(A)- mM 1_{\mathcal{K}}-\Phi\bigl(A^{2}\bigr) \bigr) - \frac{3}{4}(M-m)^{3} \widetilde{A} \\ \leq& \Phi(A)^{3} + \max_{ m_{\Phi(A)} \leq z \leq M_{\Phi(A)} } \biggl\{ \frac {(M-z)m^{3}+(z-m)M^{3}}{M-m}-z^{3} \\ &{}- 3 m(M+m) (z-M)- \frac{3}{4} (M-m)^{3} m_{\widetilde{A}} \biggr\} 1_{\mathcal{K}}. \end{aligned}$$

It suffices to put

$$\begin{aligned}& A= \frac{1}{2}\left ( \begin{matrix} -5 & -3 & 3 \\ -3 & -2 & 3 \\ 3 & 3 & 1 \end{matrix} \right ),\qquad m=-4.32147, \\& M=1.5127\quad \text{and}\quad \Phi \bigl({{ ( {{a}_{ij}} )}_{1\le i,j\le3}} \bigr)={{ ( {{a}_{ij}} )}_{1\le i,j\le2}}. \end{aligned}$$

Then (22) and (23) become

$$\begin{aligned} \frac{1}{4}\left ( \begin{matrix} -184 & -126 \\ -126 & -85 \end{matrix} \right ) \nleq&\frac{1}{8}\left ( \begin{matrix} -233 & -144 \\ -144 & -89 \end{matrix} \right ) -19.4133 I_{2} \\ &{}+71.7032 \left ( \begin{matrix} 0.116574 & -0.136402 \\ -0.136402 & 0.159602 \end{matrix} \right ) \end{aligned}$$

and

$$\begin{aligned} \frac{1}{4}\left ( \begin{matrix} -184 & -126 \\ -126 & -85 \end{matrix} \right ) < & \frac{1}{8}\left ( \begin{matrix} -233 & -144 \\ -144 & -89 \end{matrix} \right ) -19.4133 I_{2} \\ &{}+12.9644 \left ( \begin{matrix} 2.80904 & -3.28683 \\ -3.28683 & 3.84587 \end{matrix} \right ) \\ &{} -148.936 \left ( \begin{matrix} 0.116574 & -0.136402 \\ -0.136402 & 0.159602 \end{matrix} \right ) \\ < & \frac{1}{8}\left ( \begin{matrix} -233 & -144 \\ -144 & -89 \end{matrix} \right ) +76.434 I_{2}, \end{aligned}$$

respectively.

Applying Lemma 2 to a strictly convex function *f*, we improve inequalities (2) and (5).

### Theorem 3

*Let the assumptions of Lemma *2 *hold*.

*If* *f* *is a strictly convex twice differentiable on* $[m,M]$ *and* $0<\alpha\le f''$, *then*

24 $$\begin{aligned}& F \bigl[\Phi\bigl(f(A)\bigr) , g \bigl(\Phi(A) \bigr) \bigr] \\& \quad \leq \sup_{m_{\Phi(A)} \leq z \leq M_{\Phi(A)}} F \biggl[ k_{f} z +l_{f}- \frac{\alpha(M+m)z -\alpha mM -M_{1}}{2} \\& \qquad {}- \biggl( \delta_{f} - \frac{\alpha}{4}(M-m)^{2} \biggr) m_{\widetilde{A}} ,g(z) \biggr] 1_{\mathcal{K}} \\& \quad \leq \sup_{m_{\Phi(A)} \leq z \leq M_{\Phi(A)}} F \biggl[ k_{f} z +l_{f} - \biggl( \delta_{f} - \frac{\alpha}{4}(M-m)^{2} \biggr) m_{\widetilde{A}} ,g(z) \biggr] 1_{\mathcal{K}} \\& \quad \leq \sup_{m_{\Phi(A)} \leq z \leq M_{\Phi(A)}} F \bigl[ k_{f} z + l_{f} ,g(z) \bigr] 1_{\mathcal{K}}. \end{aligned}$$

### Proof

Since $(M+m)\Phi(A) -mM 1_{\mathcal{K}} -\Phi(A^{2})\geq0$ and $\alpha> 0$ gives $\alpha(M+m)\Phi(A) -\alpha mM -M_{1}\geq0$, we have

$$\begin{aligned} \Phi\bigl(f(A)\bigr) \leq& k_{f} \Phi(A) + l_{f} 1_{\mathcal{K}} -\frac{\alpha }{2} \bigl((M+m)\Phi(A) -mM 1_{\mathcal{K}} - \Phi\bigl(A^{2}\bigr) \bigr) \\ &{} - \biggl( \delta_{f} - \frac{\alpha}{4}(M-m)^{2} \biggr) \widetilde{A} \\ \leq& k_{f} \Phi(A) + l_{f} 1_{\mathcal{K}} - \frac{1}{2} \bigl( \alpha(M+m)\Phi(A) -\alpha mM -M_{1} \bigr) \\ &{}- \biggl( \delta_{f} - \frac {\alpha}{4}(M-m)^{2} \biggr) m_{\widetilde{A}}1_{\mathcal{K}} \\ \leq& k_{f} \Phi(A) + l_{f} 1_{\mathcal{K}} - \biggl( \delta_{f} - \frac {\alpha}{4}(M-m)^{2} \biggr) m_{\widetilde{A}}1_{\mathcal{K}} \\ \leq& k_{f} \Phi(A) + l_{f} 1_{\mathcal{K}}. \end{aligned}$$

Since $F(\cdot,v)$ is operator monotone in the first variable and $m_{\Phi(A)} \leq\Phi(A) \leq M_{\Phi(A)}$, we obtain (24). □

### Remark 2

We can easily generalize the above results to a bounded continuous field of self-adjoint elements in a unital $C^{*}$-algebra ${\mathcal {A}}$. Indeed, replacing *A* with $x_{t}$ and Φ with $\phi_{t}$ in (11), integrating, and using the equality $\int_{T} \phi _{t}(1_{\mathcal{H}}) \,d\mu(t)=1_{\mathcal{K}}$, we get the following inequality:

$$\begin{aligned} \int_{T}\phi_{t}\bigl(f(x_{t})\bigr) \,d \mu(t) \leq& k_{f} \int_{T}\phi_{t}(x_{t}) \,d\mu(t) + l_{f} 1_{\mathcal{K}} \\ &{} -\frac{\alpha}{2} \biggl((M+m) \int_{T}\phi _{t}(x_{t}) \,d\mu(t) -mM 1_{\mathcal{K}} - \int_{T}\phi_{t}\bigl(x_{t}^{2} \bigr) \,d\mu(t) \biggr) \\ &{}- \biggl( \delta_{f} - \frac{\alpha}{4}(M-m)^{2} \biggr) \tilde{x}. \end{aligned}$$

Next, using the operator monotonicity of $F(\cdot,v)$ in the first variable, we obtain the desired inequalities.

### Difference-type inequalities

Applying Lemma 2 to the function $F(u,v)=u- v $, we can obtain complementary inequalities to Jensen’s operator inequality for neither a convex nor a concave function *f*. These are versions of the corresponding inequalities for one operator given in [16] and [7]. We omit the details.

Next, applying this result to a convex function *f*, we obtain an improved inequality (6) and its complementary inequality for one operator.

#### Theorem 4

*Let* *A*, Φ, *Ã*, *and the bounds be as in Lemma *2.

*If* $g:[m_{\Phi(A)},M_{\Phi(A)}]\to\mathbb{R}$ *is a continuous function*, $f:[m,M]\to\mathbb{R}$ *is a strictly convex twice differentiable function*, *and* $0<\alpha\le f''$, *then*

25 $$\begin{aligned} \Phi\bigl( f( A ) \bigr) \leq& g\bigl(\Phi(A)\bigr) + \max_{m_{\Phi(A)} \leq z \leq M_{\Phi(A)}} \biggl\{ \biggl(k_{f} -\frac{\alpha}{2} (M+m) \biggr)z+ l_{f}+\frac {\alpha mM +M_{1}}{2} - g(z) \biggr\} 1_{\mathcal{K}} \\ &{} - \biggl( \delta_{f} - \frac{\alpha}{4}(M-m)^{2} \biggr) m_{\widetilde {A}} 1_{\mathcal{K}} \\ \leq& g\bigl(\Phi(A)\bigr) + \max_{m_{\Phi(A)} \leq z \leq M_{\Phi(A)}} \bigl\{ k_{f} z + l_{f} - g(z) \bigr\} 1_{\mathcal{K}} - \biggl( \delta _{f} - \frac{\alpha}{4}(M-m)^{2} \biggr) m_{\widetilde{A}} 1_{\mathcal{K}} \\ \leq& g\bigl(\Phi(A)\bigr) + \max_{m_{\Phi(A)} \leq z \leq M_{\Phi(A)}} \bigl\{ k_{f} z + l_{f} - g(z) \bigr\} 1_{\mathcal{K}} \end{aligned}$$

*and*

26 $$\begin{aligned} \Phi\bigl( f( A ) \bigr) \geq& g\bigl(\Phi(A)\bigr) + \min_{m_{\Phi(A)} \leq z \leq M_{\Phi(A)}} \bigl\{ k_{f} z + l_{f} - g(z) \bigr\} - \frac{\alpha}{2} \biggl(\frac {M-m}{2} \biggr)^{2} 1_{\mathcal{K}} \\ &{}- \biggl(\delta_{f} - \frac{\alpha}{4}(M-m)^{2} \biggr) (1-m_{\widetilde{A}})1_{\mathcal{K}} \\ \geq& g\bigl(\Phi(A)\bigr) + \min_{m_{\Phi(A)} \leq z \leq M_{\Phi(A)}} \bigl\{ k_{f} z + l_{f} - g(z) \bigr\} 1_{\mathcal{K}} - \delta_{f} (1-m_{\widetilde{A}})1_{\mathcal{K}}. \end{aligned}$$

#### Proof

Using Theorem 3, we obtain (25). Next, (26) follows from the following inequalities:

$$\begin{aligned}& \frac{\alpha}{2} \bigl(\Phi\bigl(A^{2}\bigr)+ mM 1_{\mathcal{K}}-(M+m)\Phi (A) \bigr) - \biggl( \delta_{f} - \frac{\alpha}{4}(M-m)^{2} \biggr) (1_{\mathcal{K}}-\widetilde{A}) \\& \quad \geq \frac{\alpha}{2} \bigl(\Phi(A)^{2}+ mM 1_{\mathcal {K}}-(M+m)\Phi(A) \bigr) - \biggl( \delta_{f} - \frac{\alpha }{4}(M-m)^{2} \biggr) (1-m_{\widetilde{A}})1_{\mathcal{K}} \\& \quad = \frac{\alpha}{2} \bigl(\Phi(A)-M\bigr) \bigl(\Phi(A)-m\bigr)- \biggl( \delta_{f} - \frac{\alpha}{4}(M-m)^{2} \biggr) (1-m_{\widetilde{A}})1_{\mathcal {K}} \\& \quad \geq - \frac{\alpha}{2} \biggl(\frac{M-m}{2} \biggr)^{2} 1_{\mathcal{K}} - \biggl( \delta_{f} - \frac{\alpha}{4}(M-m)^{2} \biggr) (1-m_{\widetilde{A}})1_{\mathcal{K}} \\& \quad \geq - \delta_{f} (1_{\mathcal{K}}-\widetilde{A}). \end{aligned}$$

 □

#### Remark 3

(i) Using elementary calculus, we can precisely determine the values of the constants

$$\begin{aligned}& C=\max_{m_{\Phi(A)} \leq z \leq M_{\Phi(A)}} \bigl\{ a z + b - g(z) \bigr\} , \\& c=\min _{m_{\Phi(A)} \leq z \leq M_{\Phi(A)}} \bigl\{ a z + b - g(z) \bigr\} \quad \text{for all }a,b \in\mathbb{R}, \end{aligned}$$

provided that *g* is a convex or concave function:

▶: if *g* is concave, then
  27 $$ C =\max \bigl\{ a m_{\Phi(A)}+b- g(m_{\Phi(A)}) , a M_{\Phi(A)}+b- g(M_{\Phi(A)}) \bigr\} $$
  and
  28 $$ c= \textstyle\begin{cases} {a}{{m}_{\Phi ( A )}}+{b}-g ( {{m}_{\Phi ( A )}} )&\text{if }g_{-}' ( z )\le{a}\text{ for every }z\in ( {{m}_{\Phi ( A )}},{{M}_{\Phi ( A )}} ), \\ {a}{{z}_{0}}+{b}-g ( {{z}_{0}} )&\text{if }g_{-}' ( {{z}_{0}} )\ge{a}\ge g_{+}' ( {{z}_{0}} ) \\ &\quad \text{for some }z\in ( {{m}_{\Phi ( A )}},{{M}_{\Phi ( A )}} ), \\ {a}{{M}_{\Phi ( A )}}+{b}-g ( {{M}_{\Phi ( A )}} )&\text{if }g_{+}' ( z )\ge{a}\text{ for every }z\in ( {{m}_{\Phi ( A )}},{{M}_{\Phi ( A )}} ) ; \end{cases} $$
▶: if *g* is convex, then *C* is equal to RHS in (28) with reverse inequality signs, and *c* is equal to RHS in (27) with min instead of max.

(ii) Using the same technique as in Remark 2, we can obtain generalizations of the above results for a bounded continuous field of self-adjoint elements in a unital $C^{*}$-algebra. We omit the details.

(iii) If $f\equiv g$ is strictly convex twice differentiable on $[m,M]$ and $0<\alpha\le f''$ on $[m,M]$, then (25) improves inequality in [16, Theorem 3.4]. If *f* is operator convex, then Jensen’s operator inequality holds, but if *f* is not operator convex, then (26) gives its complementary inequality.

Applying Lemma 2 and Theorem 4 to the functions $f(z)=z^{p}$ and $g(z)=z^{q}$ for selected integers *p* and *q*, we obtain the following example. These inequalities are generalizations of some inequalities in [7, Corollary 7] for nonpositive operators.

#### Example 2

Let *A* be self-adjoint operator with $\sigma ( A )\subseteq [ m,M ]$ for some $m<0<M$, $\Phi:\mathcal {B} ( \mathcal{H} )\to\mathcal{B} ( \mathcal{K} )$ be a unital positive linear mapping, *Ã* be defined by (13), and let $m_{\Phi(A)}$, $M_{\Phi(A)}$, $m_{\widetilde{A}}$, $M_{\widetilde {A}}$, $m_{1}$, and $M_{1}$ be the bounds as before.

**Figure 1:**
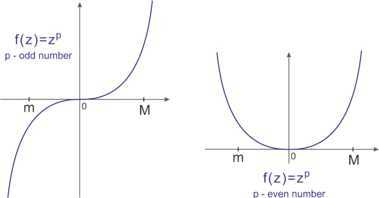
**The power function** $\pmb{f(z)=z^{p}}$ **and bounds of a nonpositive self-adjoint operator.**

(i) Let $p> 1$ be an odd number (see LHS of Figure 1), and let $q >0$ be an integer. Then $f''(z)=p(p-1)z^{p-2}$ is a monotone function. So we can take $\alpha= f''(m)$ and $\beta= f''(M)$ in Lemma 2 and obtain

29 $$\begin{aligned} \Phi\bigl( A^{p} \bigr) \leq&\Phi(A)^{q} + C^{\star}1_{\mathcal{K}} +\frac{1}{2} \bigl( M_{1}+ \alpha^{\star}mM \bigr)1_{\mathcal{K}} \\ &{} - \biggl( m^{p} +M^{p}-2^{1-p} (m+M)^{p} - \frac{\alpha^{\star}}{4}(M-m)^{2} \biggr) m_{\widetilde{A}} 1_{\mathcal{K}} \\ \leq&\Phi(A)^{q} + C^{\star}1_{\mathcal{K}} + \frac{1}{2} \bigl( M_{1}+ \alpha^{\star}mM \bigr)1_{\mathcal{K}} , \end{aligned}$$

where $\alpha^{\star}=p(p-1)m^{p-2}$,

30 $$ {{C}^{\star}}= \textstyle\begin{cases} {{k}_{p}}{{m}_{\Phi ( A )}}+{{l}_{p}}-m_{\Phi ( A )}^{q}&\text{if } { ({q}/{{{k}_{p}}} )}^{{1}/{ ( 1-q )}}\le{{m}_{\Phi ( A )}}, \\ {{l}_{p}}+ ( q-1 ){{ ({q}/{{{k}_{p}}} )}^{{q}/{ ( 1-q )}}}&\text{if }{{m}_{\Phi ( A )}}\le{{ ({q}/{{{k}_{p}}} )}^{{1}/{ ( 1-q )}}}\le {{M}_{\Phi ( A )}}, \\ {{k}_{p}}{{M}_{\Phi ( A )}}+{{l}_{p}}-M_{\Phi ( A )}^{q}&\text{if }{{ ({q}/{{{k}_{p}}} )}^{{1}/{ ( 1-q )}}}\ge{{M}_{\Phi ( A )}}, \end{cases} $$

$k_{p}:=\frac{M^{p}-m^{p}}{M-m}-\frac{\alpha^{\star}}{2} (M+m) $, and $l_{p}:=(M m^{p}-m M^{p})/(M-m)$.

Also,

31 $$\begin{aligned} \Phi\bigl( A^{p} \bigr) \geq&\Phi(A)^{q} + c^{\star}1_{\mathcal{K}} +\frac{1}{2} \bigl( m_{1}+ \alpha^{\star}mM \bigr)1_{\mathcal{K}} \\ &{} - \biggl( m^{p} +M^{p}-2^{1-p} (m+M)^{p} - \frac{\alpha^{\star}}{4}(M-m)^{2} \biggr) (1-m_{\widetilde{A}}) 1_{\mathcal{K}}, \end{aligned}$$

where

32 $$ c^{\star}=\min \bigl\{ k_{p} m_{\Phi(A)} + l_{p} - m_{\Phi(A)}^{q}, k_{p} M_{\Phi(A)} + l_{p}- M_{\Phi(A)}^{q} \bigr\} . $$

Moreover, the reverse inequalities are valid in (29) and (31) with $c^{\star}$ instead of $C^{\star}$, $m_{1}$ instead of $M_{1}$, $M_{1}$ instead of $m_{1}$, $C^{\star}$ instead of $c^{\star}$, and $\beta^{\star}=p(p-1)M^{p-2}$ instead of $\alpha^{\star}$.

(ii) Let $p> 1$ be an even number (see RHS of Figure 1), and let $q >0$ be an integer. Then $f''(z) \geq0$ is an even function. So we can put $\alpha= p(p-1) \cdot\min\{ m^{p-2}, M^{p-2} \} >0$ in Theorem 4. Applying (25) and (26), we obtain

33 $$\begin{aligned} \Phi\bigl( A^{p} \bigr) \leq& \Phi(A)^{q} + C^{\star}1_{\mathcal{K}} +\frac{1}{2} \bigl( M_{1}+ \alpha^{\star}mM \bigr)1_{\mathcal{K}} \\ &{} - \biggl( m^{p} +M^{p}-2^{1-p} (m+M)^{p} - \frac{\alpha}{4}(M-m)^{2} \biggr) m_{\widetilde{A}} 1_{\mathcal{K}} \\ \leq& \Phi(A)^{q} + C_{1}^{\star}1_{\mathcal{K}} - \biggl( m^{p} +M^{p}-2^{1-p} (m+M)^{p} - \frac{\alpha}{4}(M-m)^{2} \biggr) m_{\widetilde {A}} 1_{\mathcal{K}} \\ \leq& \Phi(A)^{q} + C_{1}^{\star}1_{\mathcal{K}} - \bigl(m^{p} +M^{p} - 2^{1-p} (m+M)^{p} \bigr) m_{\widetilde{A}} 1_{\mathcal{K}} \\ \leq& \Phi(A)^{q} + C_{1}^{\star}1_{\mathcal{K}}, \end{aligned}$$

where $C^{\star}$ and $C_{1}^{\star}$ are defined by (30) with $k_{p}:=\frac{M^{p}-m^{p}}{M-m}-\frac{\alpha^{\star}}{2} (M+m) $ and $k_{p}:=\frac{M^{p}-m^{p}}{M-m}$, respectively, and

34 $$\begin{aligned} \Phi\bigl( A^{p} \bigr) \geq& \Phi(A)^{q} + c^{\star}1_{\mathcal{K}} - \frac {\alpha}{2} \biggl(\frac{M-m}{2} \biggr)^{2} 1_{\mathcal{K}} \\ &{} - \biggl( m^{p} +M^{p}-2^{1-p} (m+M)^{p} - \frac{\alpha}{4}(M-m)^{2} \biggr) (1-m_{\widetilde{A}}) 1_{\mathcal{K}} \\ \geq& \Phi(A)^{q} + c^{\star}1_{\mathcal{K}} - \bigl( m^{p} +M^{p} - 2^{1-p} (m+M)^{p} \bigr) (1-m_{\widetilde{A}}) 1_{\mathcal{K}} , \end{aligned}$$

where $c^{\star}$ is defined by (32).

#### Remark 4

Applying Theorem 4 to strictly positive operators and the functions $f(z)=z^{p}$ and $g(z)=z^{q}$, $p,q \in\mathbb{R}$, we obtain an improvement of inequalities given in [7, Corollary 7].

Let *A* be a self-adjoint operator with $\sigma ( A )\subseteq [ m,M ]$ for some $0< m< M$.

(i) If $p \in(-\infty, 0] \cup[1,\infty)$, then (33) and (34) hold with $\alpha= p(p-1) m^{p-2} $. If $q=p \in[-1, 0] \cup[1,2] $, then the inequality $\Phi(A)^{p} \leq \Phi(A^{p})$ is tighter than (34).
(ii) If $p \in(0,1)$, then the reverse inequalities are valid in (33) and (34) with $\alpha= p(p-1) M^{p-2} $. If $q=p$, then the inequality $\Phi(A^{p}) \leq\Phi(A)^{p}$ is tighter than (33).

In all these inequalities the constants $C^{\star}$ and $c^{\star}$ are determined as follows:

▶: if $q \in(-\infty,0]\cup[1,\infty)$, then $C^{\star}$ (or $C_{1}^{\star}$) and $c^{\star}$ are defined by (30) and (32), respectively.
▶: if $q \in(0,1)$, then $C^{\star}$ (or $C_{1}^{\star}$) is equal to RHS in (32) with max instead of min and $c^{\star}$ is equal to the right side in (30) with reverse inequality signs.

### Ratio-type converse inequalities

Applying Lemma 2, similarly to the previous subsection, we can obtain complementary inequalities to Jensen’s operator inequality for neither a convex nor a concave function *f*. These are versions of the corresponding inequalities for one operator given in [16] and [7]. We omit the details.

Moreover, applying this result to a convex function *f*, we improve inequality (7) and obtain its complementary inequality for one operator.

#### Theorem 5

*Let* *A*, Φ, *Ã*, *and the bounds be as in Lemma *2.

*If* $g:[m_{\Phi(A)},M_{\Phi(A)}]\to(0,\infty)$ *is a continuous function*, $f:[m,M]\to\mathbb{R}$ *is a strictly convex twice differentiable function*, *and* $0<\alpha\le f''$, *then*

35 $$\begin{aligned} \Phi\bigl( f( A ) \bigr) \leq& \max_{m_{\Phi(A)} \leq z \leq M_{\Phi(A)}} \biggl\{ \frac { (k_{f} -\frac{\alpha}{2} (M+m) )z+ l_{f}+\frac{\alpha mM +M_{1}}{2} }{g(z)} \biggr\} g\bigl(\Phi(A)\bigr) \\ &{}- \biggl( \delta_{f} - \frac {\alpha}{4}(M-m)^{2} \biggr) m_{\widetilde{A}} 1_{\mathcal{K}} \\ \leq& \max_{m_{\Phi(A)} \leq z \leq M_{\Phi(A)}} \biggl\{ \frac {k_{f} z + l_{f}}{g(z)} \biggr\} g\bigl( \Phi(A)\bigr) - \biggl( \delta_{f} - \frac {\alpha}{4}(M-m)^{2} \biggr) m_{\widetilde{A}} 1_{\mathcal{K}} \\ \leq& \max_{m_{\Phi(A)} \leq z \leq M_{\Phi(A)}} \biggl\{ \frac {k_{f} z + l_{f}}{g(z)} \biggr\} g\bigl( \Phi(A)\bigr) \end{aligned}$$

*and*

36 $$\begin{aligned} \Phi\bigl( f( A ) \bigr) \geq& \min_{m_{\Phi(A)} \leq z \leq M_{\Phi(A)}} \biggl\{ \frac {k_{f} z + l_{f}}{g(z)} \biggr\} g\bigl(\Phi(A)\bigr) - \frac{\alpha}{2} \biggl( \frac{M-m}{2} \biggr)^{2} 1_{\mathcal{K}} \\ &{}- \biggl( \delta_{f} - \frac{\alpha}{4}(M-m)^{2} \biggr) (1-m_{\widetilde{A}})1_{\mathcal{K}} \\ \geq& \min_{m_{\Phi(A)} \leq z \leq M_{\Phi(A)}} \biggl\{ \frac {k_{f} z + l_{f}}{g(z)} \biggr\} g\bigl( \Phi(A)\bigr) - \delta_{f} (1-m_{\widetilde {A}})1_{\mathcal{K}}. \end{aligned}$$

#### Proof

We only prove the first inequality in (35). The function

$$z \mapsto\frac{ (k_{f} -\frac{\alpha}{2} (M+m) )z+ l_{f}+\frac{\alpha mM +M_{1}}{2} }{g(z)} $$

is continuous on $[m_{\Phi(A)} ,M_{\Phi(A)} ]$, and its global extremes exist. So, there are $\lambda\in \mathbb{R}$ and $z_{0}\in[m_{\Phi (A)} ,M_{\Phi(A)} ]$ such that

$$\begin{aligned} \lambda&=\max_{m_{\Phi(A)}\leq z\leq M_{\Phi(A)}} \biggl\{ \frac { (k_{f} -\frac{\alpha}{2} (M+m) )z+ l_{f}+\frac{\alpha mM +M_{1}}{2} }{g(z)} \biggr\} \\ &= \frac{ (k_{f} -\frac{\alpha}{2} (M+m) )z_{0}+ l_{f}+\frac{\alpha mM +M_{1}}{2} }{g(z_{0})}. \end{aligned}$$

Then

$$\frac{ (k_{f} -\frac{\alpha}{2} (M+m) )z+ l_{f}+\frac {\alpha mM +M_{1}}{2} }{g(z)} \leq\lambda \quad \text{for all }z\in[m_{\Phi (A)} ,M_{\Phi(A)} ]. $$

Since $g>0$, we have

$$\biggl(k_{f} -\frac{\alpha}{2} (M+m) \biggr)z+ l_{f}+ \frac{\alpha mM +M_{1}}{2} -\lambda g(z) \leq0 \quad \text{for all }z\in[m_{\Phi(A)} ,M_{\Phi(A)} ]. $$

It follows that

$$\max_{m_{\Phi(A)}\leq z\leq M_{\Phi(A)}} \biggl\{ \biggl(k_{f} - \frac{\alpha}{2} (M+m) \biggr)z+ l_{f}+\frac {\alpha mM +M_{1}}{2} -\lambda g(z) \biggr\} =0. $$

Now, applying (25) to the functions *f* and $\lambda\cdot g$, we obtain the desired inequality. □

#### Remark 5

(i) Similarly to Theorem 5, we improve inequality (8) and give its complementary inequality for one operator. For example, under the assumptions of Lemma 2, if *f* is strictly convex and twice differentiable on $[m,M]$ and $0<\alpha\le f''$, then we have

$$\begin{aligned} \Phi\bigl( f( A ) \bigr) \leq& \max_{m_{\Phi(A)} \leq z \leq M_{\Phi(A)}} \biggl\{ \frac { (k_{f} -\frac{\alpha}{2} (M+m) )z+ l_{f}+\frac{\alpha mM +M_{1}}{2} -( \delta_{f} - \frac{\alpha}{4}(M-m)^{2} ) m_{\widetilde{A}} }{g(z)} \biggr\} \\ &{}\times g\bigl(\Phi(A)\bigr) \\ \leq& \max_{m_{\Phi(A)} \leq z \leq M_{\Phi(A)}} \biggl\{ \frac{ k_{f} z + l_{f} - \delta_{f} m_{\widetilde{A}} }{g(z)} \biggr\} g\bigl( \Phi(A)\bigr). \end{aligned}$$

(ii) Using elementary calculus, we can precisely determine the values of the constants

$$K=\max_{m_{\Phi(A)} \leq z \leq M_{\Phi(A)}} \biggl\{ \frac{a z + b }{g(z)} \biggr\} , k=\min _{m_{\Phi(A)} \leq z \leq M_{\Phi(A)}} \biggl\{ \frac{a z + b }{g(z)} \biggr\} \quad \text{for every }a,b \in \mathbb{R}, $$

provided that *g* is a convex or concave function:

▶: if $g>0$ is concave, then
  37 $$ K =\max \biggl\{ \frac{a m_{\Phi(A)}+ b}{g(m_{\Phi(A)})} , \frac{a M_{\Phi(A)}+ b}{g(M_{\Phi(A)})} \biggr\} $$
  and
  38 $$ k= \textstyle\begin{cases} \frac{a m_{\Phi(A)}+ b}{g(m_{\Phi(A)})}& \text{if }g_{-}' ( z )\ge\frac{a g ( z )}{a z+b} \text{ for every }z\in ( {{m}_{\Phi ( A )}},{{M}_{\Phi ( A )}} ), \\ \frac{a {{z}_{0}}+b}{g ( {{z}_{0}} )}& \text{if }g_{-}' ( {{z}_{0}} )\le\frac{a g ( {{z}_{0}} )}{a {{z}_{0}}+b}\le g_{+}' ( {{z}_{0}} ) \text{ for some }z\in ( {{m}_{\Phi ( A )}},{{M}_{\Phi ( A )}} ), \\ \frac{a {{M}_{\Phi ( A )}}+b}{g ( {{M}_{\Phi ( A )}} )}& \text{if }g_{+}' ( z )\le \frac{a g ( z )}{a z+b} \text{ for every }z\in ( {{m}_{\Phi ( A )}},{{M}_{\Phi ( A )}} ) ; \end{cases} $$
▶: if $g>0$ is convex, then *K* is equal to RHS in (38) with reverse inequality signs, and *k* is equal to RHS in (37) with min instead of max.

(iii) If $f\equiv g$ is strictly convex twice differentiable on $[m,M]$ and $0<\alpha\le f''$ on $[m,M]$, then (35) improves [7, Theorem 12, ineq. (37)]. If *f* is an operator convex function, then Jensen’s operator inequality is tighter than (36). Otherwise, (36) gives complementary inequality to (36).

Applying Lemma 2 and Theorem 5 to the functions $f(z)=z^{p}$ and $g(z)=z^{q}$ for selected integers *p* and *q*, we can obtain inequalities of ratio type similar to Example 2. If we put $p=q$, then we can obtain generalizations of some inequalities in [7, Corollary 13] for nonpositive operators. We omit the details.

Applying Theorem 5 to strictly positive operators and the functions $f(z) \equiv g(z)=z^{p}$, we obtain the following corollary, which improves [7, Corollary 13] and [17, Corollary 3.2].

#### Corollary 6

*Let* *A* *be a self*-*adjoint operator with* $\sigma ( A )\subseteq [ m,M ]$ *for some* $0< m< M$, *and let* Φ *and the bounds be as before*. *If* $p \in(0,1)$, *then* $\Phi(A^{p}) \leq\Phi(A)^{p}$, *and if* $p \in[-1, 0] \cup[1,2] $, *then* $\Phi(A)^{p} \leq\Phi(A^{p})$. *However*, *if* $p \in(-\infty, -1) \cup(2,\infty)$ *and* $\alpha= p(p-1) m^{p-2} $, *then*

$$\begin{aligned} \Phi\bigl( A^{p} \bigr) &\leq K_{1}^{\star}\Phi(A)^{p} - \biggl( m^{p} +M^{p}-2^{1-p} (m+M)^{p} - \frac{\alpha}{4}(M-m)^{2} \biggr) m_{\widetilde{A}} 1_{\mathcal{K}} \\ &\leq K^{\star}\Phi(A)^{p} - \biggl( m^{p} +M^{p}-2^{1-p} (m+M)^{p} - \frac {\alpha}{4}(M-m)^{2} \biggr) m_{\widetilde{A}} 1_{\mathcal{K}} \\ &\leq K^{\star}\Phi(A)^{p}, \end{aligned}$$

*where*

39 $$ K^{\star}= \textstyle\begin{cases} \frac{k_{p} m_{\Phi(A)} + l_{p}}{m_{\Phi(A)}^{p}} &\textit{if } \frac{p l_{p} }{m_{\Phi(A)}}\geq(1-p) k_{p}, \\ K(m,M,p) &\textit{if } \frac{p l_{p} }{m_{\Phi(A)}} < (1-p) k_{p} < \frac{p l_{p} }{M_{\Phi(A)}}, \\ \frac{k_{p} M_{\Phi(A)} + l_{p}}{M_{\Phi(A)}^{p}} &\textit{if } \frac{p l_{p} }{M_{\Phi(A)}} \leq(1-p) k_{p}, \end{cases} $$

$k_{p}:=(M^{p}-m^{p})/(M-m)$, $l_{p}:=(M m^{p}-m M^{p})/(M-m)$, *and* $K(m,M,p)$ *is the well*-*known Kantorovich constant* (*see* [3, *Section *2.7]):

$$K(m,M,p):= \frac{m M^{p} - M m^{p} }{(p-1) (M-m)} \biggl( \frac{p-1}{p} \frac{M^{p} - m^{p}}{m M^{p} - M m^{p}} \biggr)^{p},\quad p \in \mathbb{R}. $$

*The constant* $K_{1}^{\star}$ *is calculated by* (39) *replacing* $k_{p}$ *with* $k_{p} -\frac{\alpha}{2} (M+m)$ *and* $l_{p}$ *with* $l_{p}+\frac {\alpha mM +M_{1}}{2}$.

## Quasi-arithmetic mean

As a continuation of our previous considerations, we study the order between quasi-arithmetic operator means defined by

40 $$ \mathcal{M}_{\varphi} \equiv\mathcal{M}_{\varphi}(x,{ \Phi}) := \varphi^{-1} \biggl( \int_{T} \Phi_{t} \bigl( \varphi(x_{t}) \bigr) \,d\mu(t) \biggr), $$

where $(x_{t})_{t\in T}$ is a bounded continuous field of self-adjoint operators in a $C^{*}$-algebra $\mathcal{B}(\mathcal{H})$ with spectra in $[m,M]$ for some scalars $m< M$, $(\phi_{t})_{t\in T}$ is a unital field of positive linear mappings $\phi_{t}:\mathcal{B}(\mathcal{H})\to\mathcal{B}(\mathcal{K}) $, and $\varphi\in {\mathcal{C}} ([m,M])$ is a strictly monotone function.

There is an extensive literature devoted to quasi-arithmetic means; see, for example, [3, 18–29].

First, we recall the operator order between quasi-arithmetic means (see e.g. [27, Theorem 2.1]): *Let* $(x_{t})_{t\in T}$*,* $(\phi_{t})_{t\in T}$ *be as in the definition of the quasi-arithmetic mean* (*40*)*, and let* $\psi, \varphi\in{\mathcal{C}} ([m,M])$ *be strictly monotone functions. Then*

41 $$ \mathcal{M}_{\varphi} (x,{\Phi}) \leq\mathcal{M}_{\psi} (x,{\Phi}), $$

*provided that*

(i) $\psi\circ\varphi^{-1}$ *is operator convex, and* $\psi^{-1}$ *is operator monotone, or*
(ii) $\psi\circ\varphi^{-1}$ *is operator concave, and* $-\psi^{-1}$ *is operator monotone, or*
(iii) $\varphi^{-1}$ *is operator convex, and* $\psi^{-1}$ *is operator concave*.

The order (41) without operator convexity or operator concavity is given in [27, Theorem 3.1]) under spectra conditions. In [30], some techniques are used while manipulating some inequalities related to continuous fields of operators.

Complementary inequalities to (41) are observed in [27]. We give a general result, which is tighter than that given in [27, Theorem 2.2]: *Let* $(x_{t})_{t\in T}$*,* $(\phi _{t})_{t\in T}$*,* *m, and* *M* *be as in the definition of the quasi-arithmetic mean* (*40*)*, let* $\psi, \varphi\in{\mathcal{C}} ([m,M])$ *be strictly monotone functions, and let* $F:[m,M]\times[m,M]\to \mathbb{R}$ *be a bounded and operator monotone function in its first variable*.

*If* (i) $\psi\circ\varphi^{-1}$ *is convex and* $\psi^{-1}$ *is operator monotone, or* (i′) $\psi\circ\varphi^{-1}$ *is concave and* $- \psi^{-1}$ *is operator monotone, then*

42 $$\begin{aligned} F \bigl[\mathcal{M}_{\psi}(x,{\Phi}), \mathcal{M}_{\varphi}(x,{ \Phi}) \bigr] & \leq { \sup_{ m_{\varphi} \leq z \leq M_{\varphi}} } F \bigl[ \psi^{-1} \bigl( k_{\varphi,\psi} \varphi(z) + l_{\varphi ,\psi} \bigr), z \bigr] 1_{\mathcal{K}} \\ & \leq { \sup_{ m \leq z \leq M} } F \bigl[ \psi^{-1} \bigl( k_{\varphi,\psi} \varphi(z) + l_{\varphi ,\psi} \bigr), z \bigr] 1_{\mathcal{K}}. \end{aligned}$$

*where* $m_{\varphi}$ *and* $M_{\varphi}$*,* $m_{\varphi}< M_{\varphi}$*, are bounds of the mean* $\mathcal{M}_{\varphi}(x,\Phi)$*, and*

43 $$ \begin{aligned} &k_{\varphi,\psi} := \frac{\psi(M_{\varphi})-\psi(m_{\varphi})}{\varphi(M_{\varphi})-\varphi(m_{\varphi})}, \\ &l_{\varphi,\psi} := \frac{\varphi(M_{\varphi}) \psi(m_{\varphi})-\varphi(m_{\varphi}) \psi(M_{\varphi})}{\varphi(M_{\varphi})-\varphi(m_{\varphi})}. \end{aligned} $$

Now we will study an extension and improvement of (42).

For convenience, we introduce some notation corresponding to $\delta _{f}$ in (4) and *Ã* in (13):

44 $$\begin{aligned} \begin{aligned} &\delta_{\varphi,\psi} := \psi(m_{\varphi})+\psi(M_{\varphi}) - 2 \psi \circ\varphi^{-1} \biggl( \frac{\varphi(m_{\varphi})+\varphi (M_{\varphi})}{2} \biggr), \\ &\tilde{\delta}_{\varphi,\psi} := \delta_{\varphi,\psi} - \frac {\alpha}{4} \bigl(\varphi(M_{\varphi})-\varphi(m_{\varphi})\bigr)^{2}, \\ &\tilde{x}_{\varphi} := \frac{1}{2} 1_{\mathcal{K}} - \frac {1}{|\varphi(M_{\varphi})-\varphi(m_{\varphi})|} \biggl\vert \varphi (\mathcal{M}_{\varphi} ) - \frac{\varphi(M_{\varphi})+\varphi (m_{\varphi})}{2} 1_{\mathcal{K}} \biggr\vert . \end{aligned} \end{aligned}$$

First, we give a version of Lemma 2 for means. This is an extension of (42) without convexity or concavity.

### Lemma 7

*Let* $(x_{t})_{t\in T}$, $(\phi_{t})_{t\in T}$, *m*, *and* *M* *be as in the definition of the quasi*-*arithmetic mean* (40), *let* $\psi, \varphi\in{\mathcal{C}} (I)$ *be strictly monotone functions on an interval* $I \supseteq[m,M]$, *let* $F:[m,M] \times [m,M]\to \mathbb{R}$ *be a bounded and operator monotone function in its first variable*, *and let* $m_{\varphi}$ *and* $M_{\varphi}$, $m_{\varphi}< M_{\varphi}$, *be bounds of* $\mathcal {M}_{\varphi}(x,\Phi)$.

(i) *If* $\psi\circ\varphi^{-1}$ *is a twice differentiable function such that* $\alpha\le(\psi\circ\varphi^{-1})''$ *for some* $\alpha\in \mathbb{R}$, $\psi^{-1}$ *is operator monotone*, *and*

45 $$ \begin{aligned}[b] &\sigma \biggl( \biggl( k_{\varphi,\psi}- \frac{\alpha(\varphi (M_{\varphi})+\varphi(m_{\varphi}))}{2} \biggr) \varphi(z)1_{\mathcal{K}} \\ &\quad {}+ \biggl( l_{\varphi,\psi}+ \frac{\alpha\varphi(M_{\varphi}) \varphi(m_{\varphi})}{2} \biggr) 1_{\mathcal{K}} +\frac{\alpha}{2} \varphi( \mathcal{M}_{\varphi})^{2} - \delta\tilde{x}_{\varphi} \biggr) \subseteq\psi(I) \end{aligned} $$

*for all* $z \in[m_{\varphi},M_{\varphi}]$, *then*

46 $$\begin{aligned}& F \bigl[\mathcal{M}_{\psi}(x,\Phi), \mathcal{M}_{\varphi} (x,\Phi) \bigr] \\& \quad \leq { \sup_{ m_{\varphi} \leq z \leq M_{\varphi}} } F \biggl[ \psi^{-1} \biggl( \biggl( k_{\varphi,\psi}- \frac{\alpha (\varphi(M_{\varphi})+\varphi(m_{\varphi}))}{2} \biggr) \varphi(z) \\& \qquad {}+ l_{\varphi,\psi} + \frac{\alpha\varphi(M_{\varphi}) \varphi(m_{\varphi})+\widetilde {M}_{1}}{2} - \tilde{\delta}_{\varphi,\psi} m_{\tilde{x}_{\varphi}} \biggr), z \biggr]1_{\mathcal{K}} \\& \quad \leq { \sup_{ m_{\varphi} \leq z \leq M_{\varphi}} } F \biggl[ \psi^{-1} \biggl( \biggl( k_{\varphi,\psi}- \frac{\alpha (\varphi(M_{\varphi})+\varphi(m_{\varphi}))}{2} \biggr) \varphi(z) \\& \qquad {} + l_{\varphi,\psi} + \frac{\alpha\varphi(M_{\varphi}) \varphi(m_{\varphi})+\widetilde {M}_{1}}{2} \biggr), z \biggr]1_{\mathcal{K}}, \end{aligned}$$

*where* $\widetilde{M}_{1}$ *is the upper bound of* $\alpha\varphi (\mathcal{M}_{\varphi})^{2}$, *and* $m_{\tilde{x}_{\varphi}}$ *is the lower bound of* $\tilde{x}_{\varphi}$.

*If*, *in addition*,

47 $$ \begin{aligned}[b] &\sigma \biggl( \biggl( k_{\varphi,\psi}- \frac{\alpha(\varphi (M_{\varphi})+\varphi(m_{\varphi}))}{2} \biggr) \varphi(z)1_{\mathcal{K}} \\ &\quad {}+ \biggl( l_{\varphi,\psi} + \frac{\alpha\varphi(M_{\varphi}) \varphi(m_{\varphi})}{2} \biggr) 1_{\mathcal{K}} +\frac{\alpha}{2} \varphi(\mathcal{M}_{\varphi})^{2} - \delta(1-\tilde{x}_{\varphi}) \biggr) \subseteq \psi(I) \end{aligned} $$

*for all* $z \in[m_{\varphi},M_{\varphi}]$ *then*

48 $$ \begin{aligned}[b] &F \bigl[\mathcal{M}_{\psi}(x,\Phi), \mathcal{M}_{\varphi} (x,\Phi) \bigr] \\ &\quad \geq { \inf_{ m_{\varphi} \leq z \leq M_{\varphi}} } F \biggl[ \psi^{-1} \biggl( \biggl( k_{\varphi,\psi}- \frac{\alpha (\varphi(M_{\varphi})+\varphi(m_{\varphi}))}{2} \biggr) \varphi(z) \\ &\qquad {}+ l_{\varphi,\psi} + \frac{\alpha\varphi(M_{\varphi}) \varphi(m_{\varphi})+\widetilde {m}_{1}}{2} - \tilde{\delta}_{\varphi,\psi} (1-m_{\tilde{x}_{\varphi}}) \biggr), z \biggr]1_{\mathcal{K}} \\ &\quad \geq { \inf_{ m_{\varphi} \leq z \leq M_{\varphi}} } F \biggl[ \psi^{-1} \biggl( \biggl( k_{\varphi,\psi}- \frac{\alpha (\varphi(M_{\varphi})+\varphi(m_{\varphi}))}{2} \biggr) \varphi(z) \\ &\qquad {} + l_{\varphi,\psi} + \frac{\alpha\varphi(M_{\varphi}) \varphi(m_{\varphi})+\widetilde {m}_{1}}{2} - \delta_{\varphi,\psi} (1-m_{\tilde{x}_{\varphi}}) \biggr), z \biggr]1_{\mathcal{K}}, \end{aligned} $$

*where* $\widetilde{m}_{1}$ *is the lower bound of* $\alpha\varphi (\mathcal{M}_{\varphi})^{2}$.

(ii) *If* $(\psi\circ\varphi^{-1})'' \le\beta$ *for some* $\beta\in \mathbb{R}$ *and* $-\psi^{-1}$ *is operator monotone*, *then* (46) *and* (48) *are valid with* *β* *instead of* *α*, $\widetilde{m}_{1}$ *instead of* $\widetilde{M}_{1}$, $\widetilde{M}_{1}$ *instead of* $\widetilde{m}_{1}$, *and* $M_{\tilde{x}_{\varphi}}$ *instead of* $m_{\tilde{x}_{\varphi}}$, *where* $M_{\tilde{x}_{\varphi}}$ *is the upper bound of* $\tilde{x}_{\varphi}$.

### Proof

We prove only case (i).

Replacing *A* with $\varphi(\mathcal{M}_{\varphi})$ and Φ with the identical mapping in (11), we obtain

$$\begin{aligned} \psi(\mathcal{M}_{\psi}) \leq& \frac{\psi(M_{\varphi}) - \psi (\mathcal{M}_{\varphi}) }{\varphi(M_{\varphi})-\varphi(m_{\varphi})} \psi(m_{\varphi}) + \frac{\psi(\mathcal{M}_{\varphi}) - \psi (m_{\varphi})}{\varphi(M_{\varphi})- \varphi(m_{\varphi})} \psi (M_{\varphi}) \\ &{} - \frac{\alpha}{2} \bigl( \bigl( \varphi ( {{M}_{\varphi}} )+\varphi ( {{m}_{\varphi}} ) \bigr)\varphi ( {{\mathcal{M}}_{\varphi}} )- \varphi ( {{M}_{\varphi}} )\varphi ( {{m}_{\varphi}} ){{1}_{\mathcal{K}}}- \varphi{{ ( {{\mathcal{M}}_{\varphi}} )}^{2}} \bigr) \\ &{} - \biggl( {{\delta}_{\varphi,\psi}} - \frac{\alpha}{4}{{ \bigl( \varphi ( {{M}_{\varphi}} )-\varphi ( {{m}_{\varphi }} ) \bigr)}^{2}} \biggr){{\tilde{x}}_{\varphi}}. \end{aligned}$$

Next, applying the operator monotonicity of $\psi^{-1}$ and taking into account (45), we obtain

$$\begin{aligned} \mathcal{M}_{\psi} \leq& \psi^{-1} \biggl( k_{\varphi,\psi} \psi(\mathcal{M}_{\varphi})+l_{\varphi,\psi} \\ &{}- \frac{\alpha}{2} \bigl( \bigl( \varphi ( {{M}_{\varphi}} )+\varphi ( {{m}_{\varphi}} ) \bigr)\varphi ( {{\mathcal{M}}_{\varphi}} )- \varphi ( {{M}_{\varphi}} )\varphi ( {{m}_{\varphi}} ){{1}_{\mathcal{K}}}- \varphi{{ ( {{\mathcal {M}}_{\varphi}} )}^{2}} \bigr) - \tilde{\delta}_{\varphi ,\psi} \tilde{x}_{\varphi} \biggr). \end{aligned}$$

Now, by the operator monotonicity of $F(\cdot,v)$, since $m 1_{\mathcal{K}} \leq m_{\varphi} 1_{\mathcal{K}} \leq\mathcal {M}_{\varphi} \leq M_{\varphi} 1_{\mathcal{K}} \leq M 1_{\mathcal {K}}$, we obtain the desired sequence of inequalities (46). □

### Example 3

We can apply Lemma 7 for the functions $\varphi(z)=\sin z$ and $\psi(z)=\mathrm{e}^{z}$ and one operator. We denote the appropriate means by

$$\mathcal{M}_{\mathrm{sin}} (A,\Phi) = \arcsin\bigl(\Phi(\sin A)\bigr)\quad \mbox{and} \quad \mathcal{M}_{\mathrm{e}} (A,\Phi)= \ln\bigl(\Phi\bigl( \mathrm{e}^{A}\bigr)\bigr), $$

where *A* is a self-adjoint operator with $\sigma ( A )\subseteq [ m,M ]$ for some $-\frac{\pi}{2}< m<-\frac{1}{\sqrt {2}}$, $0< M<\frac{\pi}{2}$, and $\Phi:\mathcal{B} ( \mathcal {H} )\to\mathcal{B} ( \mathcal{K} )$ is a unital positive linear mapping. Let $m_{s}$ and $M_{s}$, $m_{s} \leq M_{s}$, be the bounds of the mean $\mathcal{M}_{\mathrm{sin}}$, and let *Ã* be defined by (13).

The function $\psi\circ\varphi^{-1}(z)=\mathrm{e}^{\arcsin z}$ is neither convex nor concave on $[-1,1]$, but the function $(\psi\circ\varphi^{-1} )''(z)=\mathrm{e}^{\arcsin z} \frac {z+\sqrt{1-z^{2}}}{\sqrt{(1-z^{2})^{3}}}$ is monotone increasing. So we can put $\alpha= (\psi\circ\varphi^{-1} )'' ( \sin m_{s} )$ and $\beta= (\psi\circ\varphi^{-1} )'' ( \sin M_{s} )$.

Applying Lemma 7 and using a simple operator account, we can obtain a ratio-type order or difference-type order between these means.

Applying Lemma 7 to a strictly convex function $\psi\circ \varphi^{-1}$, we obtain improvements of inequality (42) and appropriate inequalities in [26]. We omit the proof.

### Theorem 8

*Let the assumptions of Lemma *7 *hold*.

(i) *If* $\psi\circ\varphi^{-1}$ *is a strictly convex twice differentiable function*, $0<\alpha\le(\psi\circ\varphi^{-1})''$ *for some* $\alpha \in\mathbb{R}$, $\psi^{-1}$ *is operator monotone*, *and* (45) *holds*, *then*

49 $$\begin{aligned}& F \bigl[\mathcal{M}_{\psi}(x,\Phi), \mathcal{M}_{\varphi} (x,\Phi) \bigr] \\& \quad \leq { \sup_{ m_{\varphi} \leq z \leq M_{\varphi}} } F \biggl[ \psi^{-1} \biggl( \biggl( k_{\varphi,\psi}- \frac{\alpha (\varphi(M_{\varphi})+\varphi(m_{\varphi}))}{2} \biggr) \varphi(z) \\& \qquad {}+ l_{\varphi,\psi} + \frac{\alpha\varphi(M_{\varphi}) \varphi(m_{\varphi})+\widetilde {M}_{1}}{2} - \tilde{\delta}_{\varphi,\psi} m_{\tilde{x}_{\varphi}} \biggr), z \biggr]1_{\mathcal{K}} \\& \quad \leq { \sup_{ m_{\varphi} \leq z \leq M_{\varphi}} } F \bigl[ \psi^{-1} \bigl( k_{\varphi,\psi} \varphi(z) + l_{\varphi ,\psi} - \tilde{\delta}_{\varphi,\psi} m_{\tilde{x}_{\varphi}} \bigr), z \bigr]1_{\mathcal{K}} \\& \quad \leq { \sup_{ m_{\varphi} \leq z \leq M_{\varphi}} } F \bigl[ \psi^{-1} \bigl( k_{\varphi,\psi} \varphi(z) + l_{\varphi ,\psi} \bigr), z \bigr]1_{\mathcal{K}}. \end{aligned}$$

(ii) *If* $\psi\circ\varphi^{-1}$ *is a strictly concave twice differentiable function*, $(\psi\circ\varphi^{-1})'' \le\beta<0$, *and* $-\psi^{-1}$ *is operator monotone*, *then* (49) *is valid with* *β* *instead of* *α*, $\widetilde{m}_{1}$ *instead of* $\widetilde{M}_{1}$, *and* $M_{\tilde{x}_{\varphi}}$ *instead of* $m_{\tilde{x}_{\varphi}}$.

## Results and discussion

In this paper, we obtain some complementary inequalities to Jensen’s inequality for a real-valued twice differentiable functions *f*. We obtain a generalization of known inequalities for a wider class of twice differentiable functions. Also, we obtain a refinement of some known inequalities for a class of continuous concave or convex functions. Finally, we obtain some complementary inequalities to quasi-arithmetic means with weaker conditions.

Our results have enriched the theory for the complementary inequality to Jensen’s operator inequality.

## Conclusions

Jensen’s inequality is one of the most important inequalities. It has many applications in mathematics and statistics and some other well-known inequalities are its particular cases.

This paper conducts a further study to the development of the existing theory of Jensen’s inequality for self-adjoint operators in a Hilbert space. The main contribution is the obtained complementary to Jensen’s inequality for general real-valued twice differentiable functions. The numerical examples confirm that the proposed method gives new inequalities for functions that are neither convex nor concave.

Moreover, our method gives improvements of inequalities given in [10–13] for convex or concave functions. The conditions in this paper are weaker than those in the previous research.

Finally, using the same method, we obtained new inequalities with quasi-arithmetic means. For further research, we should study improved inequalities given in [27].
